# Supplementary material for: A Tight Interaction between the Native Seagrass Cymodocea nodosa and the Exotic Halophila stipulacea in the Aegean Sea Highlights Seagrass Holobiont Variations
Source: Plants (Basel). 2023 Jan 11;12(2):350. doi: 10.3390/plants12020350 (PMC9863530; doi:10.3390/plants12020350)
Supplement: Supplementary file 1 [file plants-12-00350-s001.zip › plants-2118077-supplementary.pdf]

Conte et al.

Supplementary materials

Figure S1. Rarefaction curves

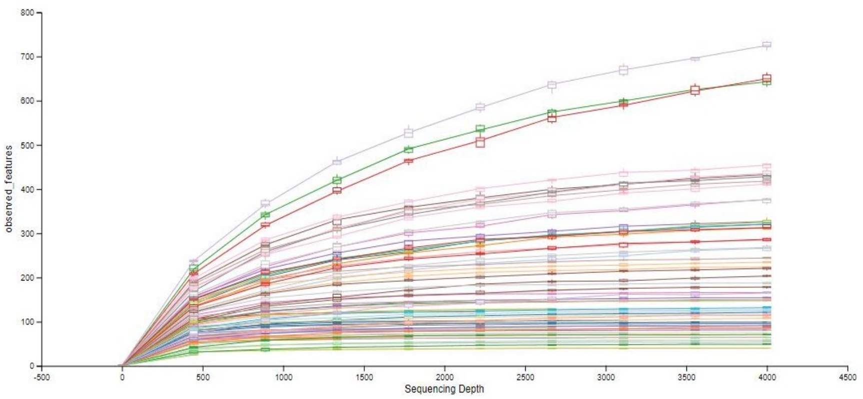

Table S1. α-Diversity as Shannon Index (H') of the microbial communities associated with both abiotic matrices (seawater and sediment) and seagrasses (leaves and roots+rhizomes) of *C. nodosa* and *H. stipulacea*) from monospecific and mixed meadows of the shallow (#1) and deep (#2) sites.

**α-Diversity**			**H' ± SD**	
**ABIOTIC MATRICES**			**SITE #1**	**SITE #2**
**Seawater**			4.0±0.1	4.6±0.0
**Sediment**	*Cymodocea nodosa*	Monospecific	5.5±0.9	4.3±0.1
	Halophila stipulacea	Monospecific	4.6±0.5	3.9±0.4
		Mixed	4.8±1.1	4.4±0.2
**SEAGRASS**				
	Plant part	***Meadow type***	**SITE #1**	**SITE #2**
C. nodosa	Aboveground	Monospecific	5.6±0.1	5.1±0.6
		Mixed	5.4±0.3	4.9±0.3
	Belowground	Monospecific	5.3±0.5	4.6±0.7
		Mixed	5.2±0.1	4.4±0.4
H. stipulacea	Aboveground	Monospecific	5.2±0.4	4.2±0.7
		Mixed	4.9±0.7	4.7±0.3
	Belowground	Monospecific	5.2±0.4	4.3±0.7
		Mixed	4.3±0.6	4.±0.5

Table S2. 2-way ANOSIM results of Biotic data comparison using the site and the meadow zone as source of variance

**Parameter**	**Source of variance**	2-way ANOVA	
		F	*p*
Meadow density	Site	23.67	$p < 0.05$
	Meadow zones	-	NS
Leaf area	Site	-	NS
	Meadow zones	8.14	$p < 0.05$
TLR	Site	63.13	$p < 0.05$
	Meadow zones	12.06	$p < 0.05$
Chl a	Site	-	NS
	Meadow zones	-	NS
Chl b	Site	4.08	$p < 0.05$
	Meadow zones	-	NS
Car	Site	-	NS
	Meadow zones	-	NS
Phenols	Site	34.35	$p < 0.05$
	Meadow zones	12.79	$p < 0.05$
TC- Leaves	Site	18.3	$p < 0.05$
	Meadow zones	229.3	$p < 0.05$
δC^{13} - Leaves	Site	232	$p < 0.05$
	Meadow zones	335.4	$p < 0.05$
TN - Leaves	Site	-	NS
	Meadow zones	-	NS
δN^{15} - Leaves	Site	7.95	$p < 0.05$

	Meadow zones	451.1	$p < 0.05$
TC- Rhizome	Site	-	NS
	Meadow zones	-	NS
δC[13] - Rhizome	Site	336.1	$p < 0.05$
	Meadow zones	640.6	$p < 0.05$
TN - Rhizome	Site	10.82	$p < 0.05$
	Meadow zones	6.58	$p < 0.05$
δN[15] - Rhizome	Site	25.93	$p < 0.05$
	Meadow zones	875.7	$p < 0.05$
TC- Roots	Site	4.35	$p < 0.05$
	Meadow zones	40.66	$p < 0.05$
δC[13] - Roots	Site	139	$p < 0.05$
	Meadow zones	112.8	$p < 0.05$
TN - Roots	Site	77.98	$p < 0.05$
	Meadow zones	49.28	$p < 0.05$
δN[15] - Roots	Site	92.9	$p < 0.05$
	Meadow zones	6.64	$p < 0.05$
